# Exploring the Adaptation of *Bulinus senegalensis* and *Bulinus umbilicatus* to the Dry and Rainy Season in Ephemeral Pond in Niakhar (Senegal), an Area of Seasonal Transmission of Urogenital Schistosomiasis

**DOI:** 10.3390/tropicalmed9060121

**Published:** 2024-05-22

**Authors:** Diara Sy, Bruno Senghor, Cheikh Sokhna, Mamadou Aliou Diallo, Amélé Nyedzie Wotodjo, Doudou Sow, Souleymane Doucoure

**Affiliations:** 1 EMR MINES: Maladies Infectieuses, Négligées et Émergentes au Sud, Institut de Recherche pour le Développement, Campus International Institut de Recherche pour le Développement-Université-Cheikh Anta Diop of Hann, Dakar BP 1386, Senegal; radiadaty@gmail.com (D.S.); bruno.senghor@yahoo.fr (B.S.); cheikh.sokhna@ird.fr (C.S.); mamadou.diallo1@ird.fr (M.A.D.); amele-nyedzie.wotodjo@ird.fr (A.N.W.); 2 EMR MINES Maladies Infectieuses, Négligées et Émergentes au Sud, Aix Marseille Université, Institut de Recherche pour le Développement, 13005 Marseille, France; 3 Department of Animal Biology, Faculty of Sciences and Techniques, Université-Cheikh Anta Diop, Dakar BP 5005, Senegal; 4 Department of Parasitology-Mycology, Unité de Formation et de Recherche des Sciences de la Santé, Université Gaston Berger, Saint-Louis BP 234, Senegal; doudsow@yahoo.fr

**Keywords:** Senegal, schistosomiasis, *Bulinus*, season, aestivation

## Abstract

*Bulinus* snails surviving drought play a key role in the seasonal transmission of urogenital schistosomiasis, although our knowledge of their adaptation to dry season is still limited. We investigated the survival dynamic and infestation by the *Schistosoma haematobium* of *Bulinus* snails during the dry and rainy seasons in a single pond in an area of seasonal schistosomiasis transmission in Senegal. During the rainy season, 98 (94.23%) *B. senegalensis* and six (5.76%) *B. umbilicatus* were collected, respectively. In the dry season, *B. umbilicatus* outnumbered *B. senegalensis*, but all five (100%) *B. senegalensis* collected were viable and alive after the interruption of aestivation by immersion in water, while only 7 of 24 (29.16%) *B. umbilicatus* collected emerged from their dormant state. The rate of infestation with *S. haeamatobium* during the rainy season was 18.2% (19/104), while all the viable snails collected during the dry season were negative. *B. senegalensis* and *B. umbilicatus* have different seasonal dynamics with no evidence of maintaining *S. haematobium* infestation during the drought. Further studies including more survey sites and taking account both snails biology and ecological conditions are needed to better understand snail adaptation to seasonal changes and their ability to maintain *S. haeamatobium* infestation during drought.

## 1. Introduction

Schistosomiasis is a neglected tropical disease (NTD) caused by blood flukes of the genus *Schistosoma*. Schistosomiasis disease is highly associated with poor sanitation and lack of clean water in endemic rural areas [1]. According to the World Health Organization, it is estimated that more than 250 million people need preventive treatment worldwide [2]. In 2019, the disability-adjusted life years (DALY) lost due to schistosomiasis was estimated at 1.6 million [3]; thus, schistosomiasis is a major human health problem and an obstacle to the economic development of populations living in rural areas [4]. In Africa, schistosomiasis is transmitted to humans by freshwater snails of the genius of *Biomphalaria* and *Bulinus*. The epidemiology of the disease is closely linked to the presence of freshwater sources representing both snails breeding sites and schistosomiasis transmission places. In areas with perennial transmission, water sources are available throughout the year, maintaining high densities of snails [5], contrasting with seasonal transmission areas characterized by a period of drought, coinciding with the dry season [6,7]. In these ecosystems, the snail vectors survive the drought thanks to their aestivation capacities and are only active during the rainy season. The adaptation of snails to drought may vary according to species and bio-ecological conditions [8,9,10,11]. This feature helps maintain snail populations during the dry season and ensures the rapid repopulation of temporary pounds at the start of the rainy season [12]. This ability gives *Bulinus* snails a pivotal role in the epidemiology of the disease in areas of seasonal transmission. Many studies have used experimental procedures to highlight the differential adaptation of snails species to drought [11,13,14]. These experimental models do not accurately reflect the natural interactions between the different species of molluscs and their environment as most of the snail strains are breed in laboratories for a long period and may therefore lose their ability to adapt to natural conditions. In addition, the variability of natural conditions can be very different from that observed under experimental conditions. Even though it is possible to investigate snail adaptation and survival in natural conditions [15], to date, studies carried out under natural conditions are rare. Hence, the mechanisms underlying the survival of *Bulinus* snails during drought and their emergence in the rainy season remain largely unexplored under natural conditions in addition to the interaction between the parasite and dormant snail. Indeed, it is thought that the snails could maintain their infestation during the dry period and become infested immediately after the first rain [7]. Understanding the aestivation mechanisms of snails and their ability to retain the parasite during diapause could improve our knowledge of the emergence and transmission dynamics of the disease in areas with temporary ponds.

We carried out a study in Niakhar in central-western Senegal, where *B. senegalensis* and *B. umbilicatus* can withstand 7–8 months of drought [7], to understand these *Bulinus* population dynamics and infestation by *S. heamatobium* both in the rainy and dry seasons and their viability after stopping their aestivation. Their ability to survive the drought was also explored experimentally.

## 2. Materials and Methods

### 2.1. Study Area and Ponds

**Figure 1:**
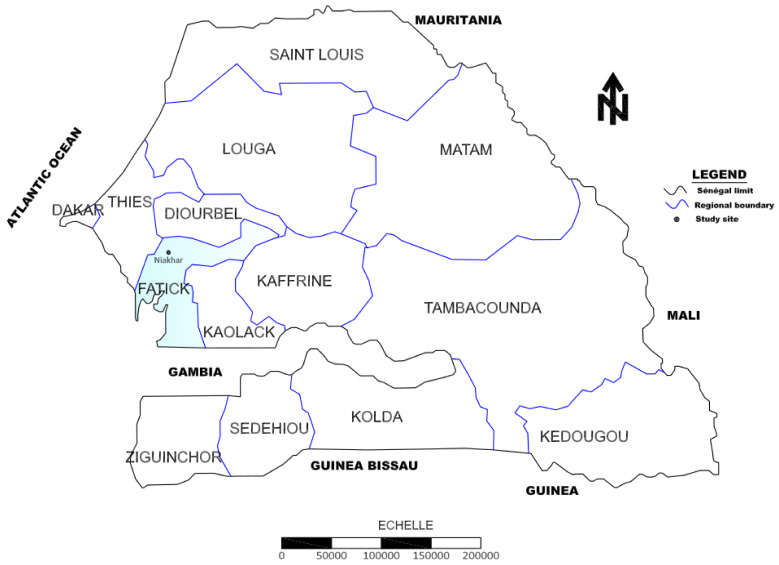
Map of Senegal showing the geographical location of the Fatick and the Niakhar study area.

The investigation was carried out at the Niakhar commune located in the in the region of Fatick, central-western Senegal. Niakhar is located 140 Km to the south-east of Dakar, the capital city (Figure 1). The area is characterized by the presence of temporary ponds where a seasonal transmission of urogenital schistosomiasis occurs during the rainy season from June/July to October [7]. 

**Figure 2:**
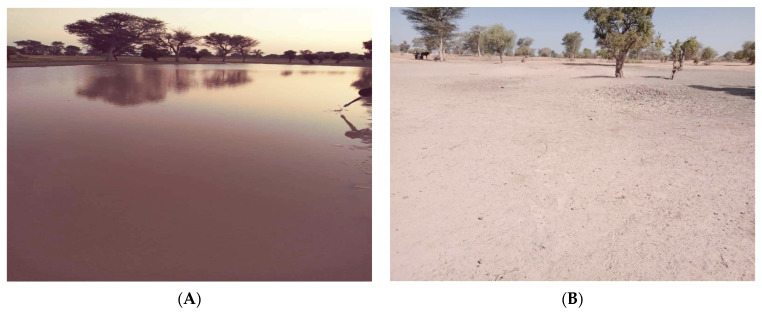
Mbel Khakhale pond at Niakhar study site. (**A**) The pond filled with water during the rainy season. (**B**) The pond dried up during the dry season.

The Niakhar area includes 40 villages of which about 17 have a relatively detailed description of the different species of snail vectors of *Schistosoma* species. *B. senegalensis* and *B. umbilicatus* are the main species involved in the transmission of *S. haematobium* [7]. Among all the ponds surveyed in the area, only the pond located in the village of Ngangarlam hosts both *B. senegalensis* and *B. umbilicatus* [7]. This pond named “Mbel Khakhale” is one of the most infested by *S. haematobium* [16]. The pond has a depth of 1 m, and it is about 50 m long and 40 m wide and has two access points and is only supplied with water during the rainy season (Figure 2).

### 2.2. Bulinus Snail Collection and Testing for Schistosoma haematobium Infection

The snails were collected during the rainy and dry seasons. They were also tested to assess their infestation by *Schistosoma haematobium* (see in the Supplementary materials, Figure S1).

- Dry season

The snails were collected in May 2022 during the dry season. The snails were prospected around the centre and in the hollow areas after the pond had dried out. At each collection point, cores were performed using a core barrel 30 cm high and 10 cm wide. The ground was examined to collect the snails at 5, 10 and 15 cm to determine the sinking capacity of the snails. 

At the same time, the entire surface of the pond was prospected to collect plastic waste, pieces of wood and even the dry bark of trees in order to collect the sand that covered them when the pond dried out. 

All soil samples were lightly broken and sieved in a basin containing water to separate the snails from small stones and other debris. Then, the recovered snails were placed in tanks containing moistened sand from the pond to gradually prepare them for waking up while they were transported to the laboratory. Snails with empty shells were excluded from the study, while those with the body retracted to the bottom of the shell were placed in water for 24 h to check their viability. A snail was considered non-viable if it remained shrunken in its shell, unable to climb the wall of the container or died less than 24 after its immersion in water.

- Rainy season

The snail collection was carried out in October 2022 at the end of the rainy season. At this time of year, the pond is still wet. The snail survey was carried out on the bank and beyond to about 2 m. The snails were then collected during 15 min by two collectors with a dip net. Then, all snails collected were placed in a plastic container filled with some aquatic plants and pond water before being transferred to the laboratory.

All snails collected during the dry and the rainy season were morphologically identified according to Brown and Kristensen’s taxonomic keys [17].

- Cercarial shedding test

Cercariae shedding test for the detection of the *Schistosoma haematobium* parasite were performed on each snail collected during the rainy season and on all viable snails recovered during the dry season. Each snail was tested, once in the laboratory, by placing it in a glass tube with 10 mL of filtered water and exposing it to direct sunlight or electric light for 45 min to 1 h to induce cercariae shedding. Schistosome cercariae shed from infested snails were then observed under a dissecting microscope and identified according to the criteria developed by Fransden and Christensen [18].

- Molecular analysis

### 2.3. DNA Extraction

The cetyl trimethyl ammonium bromide (CTAB) method was used to extract genomic DNA from the head-to-foot portion [19]. This dissected part of each snail was ground with a pestle in 200 μL of 2% filtered CTAB and the mixture was incubated at 65 °C for 1 h. Then, 200 μL of chloroform was added for the nucleic acid clean-up. The mixture was then centrifuged at 12,000 rpm for 5 min, and the supernatant was recovered, with 200 µL of isopropanol added to precipitate the DNA. After 15 min of centrifugation at 12,000 rpm, the isopropanol was poured off and the tube was drained into paper towels. The procedure was repeated with 200 μL of 70° ethanol but the centrifugation took 5 min. The purified DNA was dried using a Speed-Vac concentrator for 5 min at 45 °C and resuspended in 100 μL of pure water. The DNA extracts were stored at −20 °C until use. 

### 2.4. PCR Procedures

Two successive PCRs were performed to determine the prevalence of *S. haeamatobium*. Real-time PCR targeting the Dra1 gene was used first to highlight the presence of parasites of the *haematobium* group, followed by a standard PCR for the specific detection of *S. haematobium*.

#### 2.4.1. Real-Time PCR (RT-PCR)

All snails collected during the rainy and dry seasons were tested with real-time PCR. The Dra1 tandem repeat sequence is the target region for real-time PCR; it allows the detection of the *Schistosoma haematobium* group parasite [20,21,22]. The PCR reaction was performed with a mixture of 20 μL including 5 μL of DNA, 3.5 μL of sterile pure water, 0.5 μL of forward primer (5′-GATCTCACCTATCAGACGAAAC-3′), 0.5 μL of reverse primer (5′-TCACAACGATACGACCAAC-3′), 0.5 μL of TaqManTM probe (Applied Biosystems, Foster City, CA, USA) (5′-TGTTGGTGAAGTGCCTGTTTCGCAA-3′), and 10 μL of master mix. The thermal cycler, CFX96 (Bio-Rad C 1000 Touch, Marnes-la-Coquette, France), was used to perform the amplification. The program consisted of an initial denaturation step of 2 min at 50 °C, followed by a 3 min denaturation at 95 °C, and then 40 cycles of 95 °C for 30 s and 60 °C for 1 min before holding the sample at 4 °C. The result was considered positive if the cycle threshold (Ct) value was less than 35. In each run, positive and negative controls were performed with the samples. DNA extracts from adult *S. haematobium* worms were used as positive controls and pure water as the negative control. Then, all Dra1-positive specimens were tested for *S. haematobium* using standard PCR.

#### 2.4.2. Rapid Diagnostic PCR (RD-PCR) to Detect *S. haematobium*

Dra1 real-time PCR-positive samples were tested for *S. haematobium* by RD-PCR [23]. Amplification was performed in a 25 μL reaction volume comprising 2 μL of DNA, 12.5 μL AmpliTaq Gold^®^360 PCR Mix (Applied Biosystems™, Waltham, MA, USA), 8.5 μL of sterile distilled H_2_O, 1 μL of universal direct Asmit1 primer (TTTTTTGGTCATCCTGAGGTGTAT) and 1 μL of reverse primer (Sh. R: 5′-TGATAATCAATGACCCTGCAATAA-3′). The amplification protocol was performed with initial denaturation at 95 °C for 15 min, with 39 cycles at 95 °C for 30 s, 58 °C for 1 min, 72 °C for 1 min, and a final step at 72 °C for 7 min using a thermal cycler (Applied Biosystems, Foster City, CA, USA). Migration was performed for 1 h and 15 min at 180 V in a 1.5% agarose gel with a SYBR Safe dye and readout was performed using the Gel Doc system (Bio-Rad Universal Hood II, Hercules, CA, USA).

- Evaluation of the Bulinus survival to drought under semi-experimental conditions

**Figure 3:**
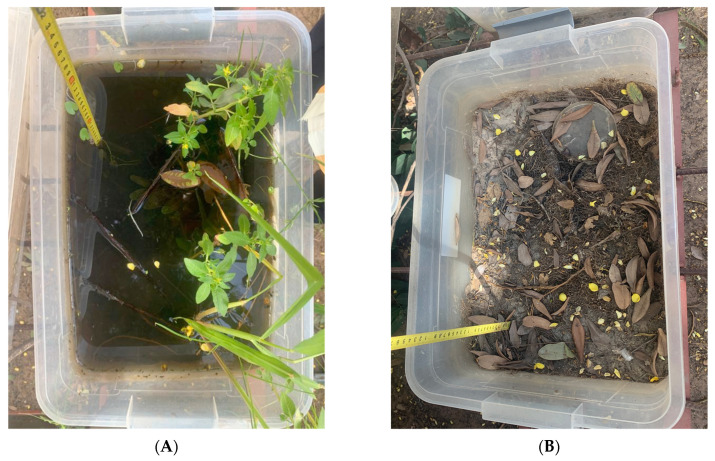
Tanks used to reproduce drought in semi-natural conditions. (**A**) Tank filled with water; (**B**) tank dried out after two months.

A semi-natural experimental device reproducing the drying of ponds was set up for this purpose (Figure 3). For the setting up of the device to reproduce the progressive drying of the ponds, two plastic tanks (“artificial ponds”) were each filled with sand from the study site to a height of 15 cm. Then, 5 L of water was added to each container before they were left to gradually dry in a period of one month. After that, the tanks were refilled with the same amount of water, and then 15 *B. senegalensis* and 8 *B. umbilicatus* specimens derived from laboratory breeding were added in each. The tanks were then left to dry out and were completely dry after 15 days. Two months after they had dried out completely, snails were recovered from the dry sand and put in water to induce the emergence of the specimens undergoing aestivation (Figure 3) and to monitor their mortality and reproduction.

### 2.5. Statistical Analysis

Fisher’s exact test was used to compare the snail population during the rainy and the dry season and also between the snail populations in semi-natural conditions. All analyses involved two-sided *p* values, with statistical significance defined by *p* ≤ 0.05.

## 3. Results

### 3.1. Snail Species Collected and Infestation Rates

- Dry season

During the dry season, a total of 29 snails were collected, of which 24 (82.75%) and 5 (17.24%) were morphologically identified as *B. umbilicatus* and *B. senegalensis*, respectively. Both species were collected from the sand using the core barrel and mostly at 5 cm of depth. However, some *B. umbilicatus* were found buried under plastic waste, while some *B. senegalensis* were collected from tree trunk bark.

After careful observation, all snail specimens had intact shells. Among the 24 *B. umbilicatus* specimens, 10 (41.66%) showed no sign of life once put in water despite the presence of their soft part, 7 (29.16%) came out of their dormant state but did not survive beyond 24 h and 7 (29.16%) were maintained alive until giving two consecutive generations. All five specimens of *B. senegalensis* survived after 24 h exposure in water and gave offspring that survived for a month.

- Rainy season

During the rainy season, 104 snails were collected. The snails were mainly collected on the leaves of the white water-lily (*Nymphaea alba*), a plant that grows in the pond during the rainy season, to which the snails tend to cling, and also on solid objects found in the pond. Of the 104 snails collected, 98 individuals (94.23%) were identified as *B. senegalensis* and 6 individuals (5.76%) as *B. umbilicatus*.

**Figure 4:**
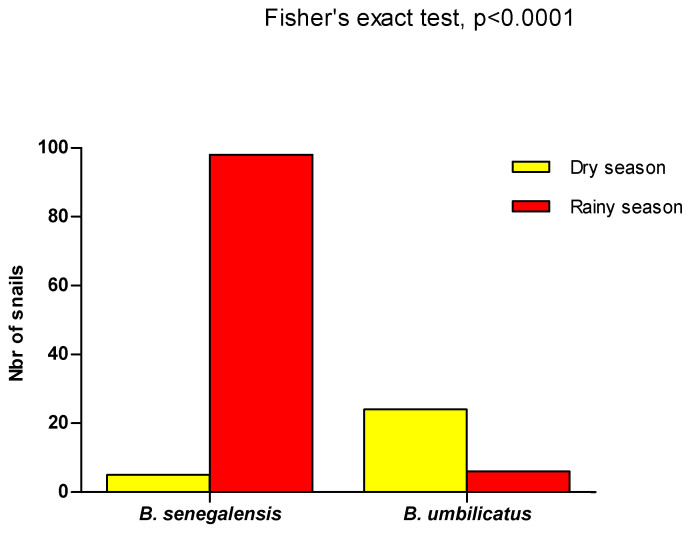
Distribution of *B. senegalensis* and *B. umbilicatus* in the wet and dry seasons. A total of 5 *B. senegalensis* and 24 *B. umbilicatus* were found during the dry season. During the rainy season, 96 *B. senegalensis* and 4 *B. umbilicatus* specimens were found. In the dry season, *B. umbilicatus* is the dominant species compared to *B. senegalensis*, while the reverse is observed during the rainy season (Fisher’s exact test, *p* < 0.0001).

The presence of *B. senegalensis* was significantly important compared with *B. umbilicatus* during the rainy season, while during the dry season, *B. umbilicatus* was the predominant species (Fisher’s exact test, *p* < 0.0001) (Figure 4).

### 3.2. Snail Infestation Rates

All viable snails collected during the dry season, seven *B. umbilicatus* and five *B. senegalensis*, were found to be negative upon cercarial emissions and molecular tests. Of the 104 snails collected during the rainy season, 29.80% (31/104) emitted *Schistosoma* cercariae. Among the 31 snails found to be positive using the *Schistosoma* cercariae shedding test, 90.32% (28/31) and 9.76% (3/31) were identified as *B. senegalensis* and *B. umbilicatus*, respectively. However, a total positivity rate of 35.5% (37/104) was obtained using Dra1 RT-PCR. Among the 37 specimens positive to Dra1 RT-PCR, 32 and 5 were identified as *B. senegalensis* and *B. umbilicatus*, respectively. The use of RD-PCR showed that among these Dra1-positive specimens, 50% (16/32) and 60% (3/5) of *B. senegalensis* and *B. umbilicatus*, respectively, were positive to *S. haematobium* DNA. Considering both species, the positivity rate of *S. haematobium* was 18.2% (19/104) in the pond prospected in October in the rainy season.

### 3.3. Evaluation of the Bulinus Survival to Drought under Semi-Experimental Conditions

**Figure 5:**
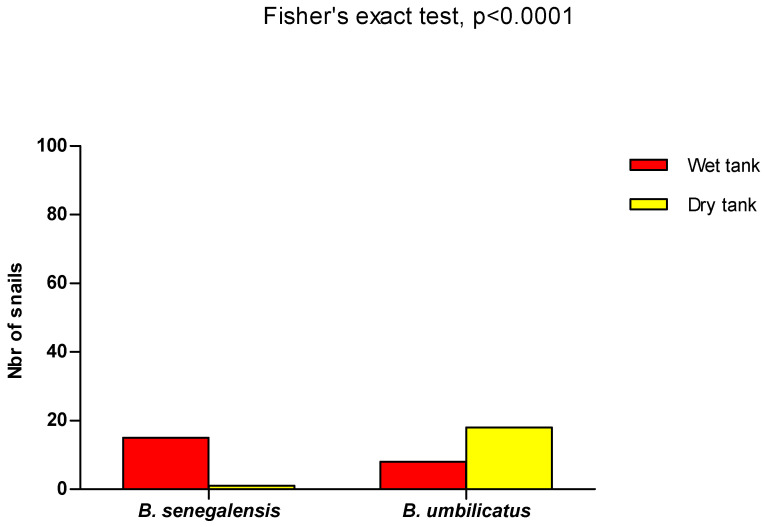
*B. senegalensis* and *B. umbilicatus* in dry and wet reservoirs. Of the 15 initial *B. senegalensis* (wet reservoir), only 1 was found alive after the reservoir had dried out. For *B. umbilicatus*, there were 8 and 18 specimens alive before (wet reservoir) and after (dry reservoir), respectively. *B. senegalensis* was more dominant compared with *B. umbilicatus* in the wet tank, whereas *B. umbilicatus* was dominant in the dry tank (Fisher’s exact test, *p* < 0.0001).

In the artificial ponds, after they had been drained and re-watered, only 1 specimen of the original population of 15 *B. senegalensis* was found. For *B. umbilicatus*, 18 specimens were found, whereas the initial population was 8 individuals. All the species recovered were placed in tanks filled with water in order to monitor their development. After 15 days of observation, no mortality was recorded. Eggs and juveniles were recorded in the tank containing the specimen of *B. senegalensis*, whereas in the tank containing the species of *B. umbilicatus*, there were no eggs. The presence of *B. senegalensis* was significantly dominant compared with *B. umbilicatus* in the wet tank, while the opposite trend was observed when the tank dried (Fisher’s exact test, *p* < 0.0001) (Figure 5).

## 4. Discussion

In the Niakhar area, located in central Senegal, schistosomiasis transmission occurs seasonally in temporary ponds. In this area, snail populations are highly dependent on rainfall and enter aestivation during the dry season [7]. In the course of this study, we reported on snail dynamics in a single pond at Niakhar during the rainy and the dry season. Our results, reporting the presence of *B. senegalensis* and *B. umbilicatus*, confirm previous work carried out during the rainy season in the same pond [7,16]. In addition, for the first time, we found, in the Niakhar area, the presence of both species during the dry season when the pond was totally dry. During the dry season, *Bulinus* specimens were not only collected exclusively in the dry mud but also in plastic residues covered with a layer muddy sand and plant waste. It would be interesting to assess the importance of these solid objects in the survival of snails when the ponds dry out and to see to what extent clean-up measures can be taken. This possibility of using clean-up action to eliminate drought-surviving snails would be an advantage over the use of chemicals during the rainy season when human activity is very high. In addition, it could be possible to raise public awareness of the risks of pond pollution on schistosomiasis transmission. However, more extensive studies, including more sites, would be needed to see whether plant and plastic debris would serve as significant refuges for snails, as most studies have reported that molluscs tend to bury themselves in the mud to survive drought, although some work has shown the possibility of molluscs burrowing between the roots of water lilies when ponds dry out [15] and also preferring the upper periphery of the gites [24].

During the rainy season, *B. senegalensis* was the dominant species compared to *B. umbilicatus*, and this trend is usually observed in Ngangarlam pond at Niakhar where these two *Bulinus* species are sympatric [7,16,25]. However, in the south-east of Senegal, it has been shown that the density of *B. umbilicatus* can be much higher than that of *B. senegalensis* in ephemeral ponds [15], suggesting that ecological conditions influence the density of different mollusc species on a local scale. In Niakhar, previous studies carried out during the rainy season have shown that *B. snegalensis* was the dominant species [7,16]. This trend is not observed during the dry season when *B. umbilicatus* outnumbered *B. senegalensis*. Thus, depending on the season, it is possible to have two dynamics linked to the ability of each species to survive the drought or the re-watering of the ponds. In the Niger Sahelian region, *B. senegalensis* and *B. truncates*, with very different aestivation capacities, have different population dynamics in persistent ponds filled with water for more than six months [26].

Based on the number of snails collected in the field during the dry season, it seems that *B. umbilicatus* is better adapted to drought than *B. senegalensis*. A previous study in south-eastern Senegal showed a higher density of *B. umbilicatus* compared with *B. senegalensis* just after the first rains of the season. This could mean that they predominate throughout the dry season [15]. Moreover, in the Gambia, in the Soudano-Sahelien area, a decrease in the survival of *B. sengalensis* in aestivation was observed over time during the dry period [27]. This trend was also observed in our semi-natural experiment, where *B. umbilicatus* was mainly found after the tanks had dried out, even though we used laboratory specimens which may behave differently from wild isolates. But, an extensive prospection should be conducted to gather data in other ponds and sites as *B. senegalensis* is described to be well adapted to drought [27]. The fact that there were more *B. senegalensis* during the rainy season could be explained by the greater capacity of this species to end its aestivation as soon as the rains return and its short generation time [28]. 

During this study, we observed a higher mortality rate in *B. umbilicatus* from natural conditions returned to water, whereas no mortality was observed in *B. senegalensis*. This could indicate that these two species do not have the same resilience to drought and to the pond re-watering and could reveal the complex dynamics of snails in temporary waterholes. In order to elucidate this complexity, more ecological and biological parameters need to be considered. Indeed, as ponds dry out, the increase in soil temperature can affect the ability of land snails to regulate their body temperature, and this is more pronounced in smaller snail species [29]. It is therefore likely that *B. senegalensis*, which is smaller than *B. umbilicatus*, is more sensitive to the increase in temperature during drought. At the species level, it is also well established that medium-sized molluscs are more resistant to drought [7,15,30]. However, during this study, we did not measure the size of the specimens to assess whether this plays a role in the survival of both *B. umbilicatus* and *B. senegalensis* during the dry season. 

Also, the depth of their burial in the mud, the presence of vegetation, the water saturation of the mud at the time of burial, and soil quality combined with the anatomical specificities of each species may influence their aestivation capacity [27,31]. Therefore, combining experimental and field studies could help to better understand both the biological and ecological parameters influencing snails survival during the dry season. In our study, we observed the same trends between natural and semi-natural collections, indicating that it is possible to develop studies in controlled environments to understand the adaptation of molluscs to their changing environment. However, it is important to note that we used sand from natural ponds and recently colonised mollusc strains.

Previous studies carried out in the same pond showed a higher Dra1 positivity rate than that observed in this study [16]. However, the positivity rate of *S. haematobium* is currently higher than in collections made in 2015, where 2 out of 98 snails were positive in the molecular test [32]. This could be explained by the fact that in 2015, the snails were collected after praziquantel mass administration. During this study, none of the snail collected in the dry season were harbouring schistosome parasites despite the use of sensitive molecular techniques compared to the cercariae shedding method. In areas with seasonal transmission, it is assumed that the molluscs retain their infestation during the dry season and become infested as soon as the rains return [33]. In the Niakhar area, the possibility of *B. umbilicatus* maintaining its infestation for a period of 7 months of drought was highlighted [7]. In our study, although we used cercarial emission and molecular biology techniques, we did not find any molluscs infested during the drought. This could be due to the low number of molluscs collected during this season. This represents a limitation to this study, and a larger sampling including more ponds in diverse ecological areas would certainly be needed to determine the capacity of the snails to maintain the infestation during the dry seasons. In fact, compared with other studies, we did not test the snails at the beginning of the rainy season to assess their capacity to retain the parasite during the dry season. The beginning of the rainy season could be more favourable for finding more molluscs that have resisted the drought. On the other hand, it should also be borne in mind that infested molluscs may be more susceptible to desiccation than healthy molluscs, which could contribute to a reduction in their population during the dry season [34]. In addition the parasite survival is reduced during aestivation under experimental conditions [35]. However, in this study, we were only interested in *S. haematobium*. The possibility of snail infestation by non-*S. haematobium* was not evaluated. The presence of *S. bovis* and hybrids between *S. haematobium* and *S. bovis* have been highlighted in *B. senegalensis* in the pond we investigated [32]. A complete and specific detection non-*S. haematobium* infestation is therefore necessary to better understand the survival of snails and the parasites they harbour. In any case, a better understanding of the survival mechanisms of molluscs during the dry season should help us to understand the epidemiological system and the dynamics of infestation of human populations in areas of the seasonal transmission of schistosomiasis.

## 5. Conclusions

This study shows that the dynamics of *B. senegalensis* and *B. umbilicatus* could be season- and species-dependent in ephemeral ponds. A better understanding of this differential adaptation between sympatric species could provide new insights into the role of *Bulinus* snails in seasonal transmission systems for bilharzia. The combination of experimental and field studies would provide relevant information on the complexity of the *Bulinus* snail’s development cycle in areas of seasonal transmission. The specific ecological conditions offered by dried-up ponds could represent a breakthrough for snail control.

## Data Availability

The datasets used and/or analysed during the current study are available from the corresponding author on reasonable request.

## Supplementary Materials

The following supporting information can be downloaded at: https://www.mdpi.com/article/10.3390/tropicalmed9060121/s1, Figure S1: Description of the procedure used to collect molluscs during the dry and rainy seasons, to assess their viability and their infestation by *S. haematobium*.
