# Supplementary material for: Exploring the Adaptation of Bulinus senegalensis and Bulinus umbilicatus to the Dry and Rainy Season in Ephemeral Pond in Niakhar (Senegal), an Area of Seasonal Transmission of Urogenital Schistosomiasis
Source: Trop Med Infect Dis. 2024 May 22;9(6):121. doi: 10.3390/tropicalmed9060121 (PMC11209171; doi:10.3390/tropicalmed9060121)
Supplement: Supplementary file 1 [file tropicalmed-09-00121-s001.zip › tropicalmed-2946759-supplementary.pdf]

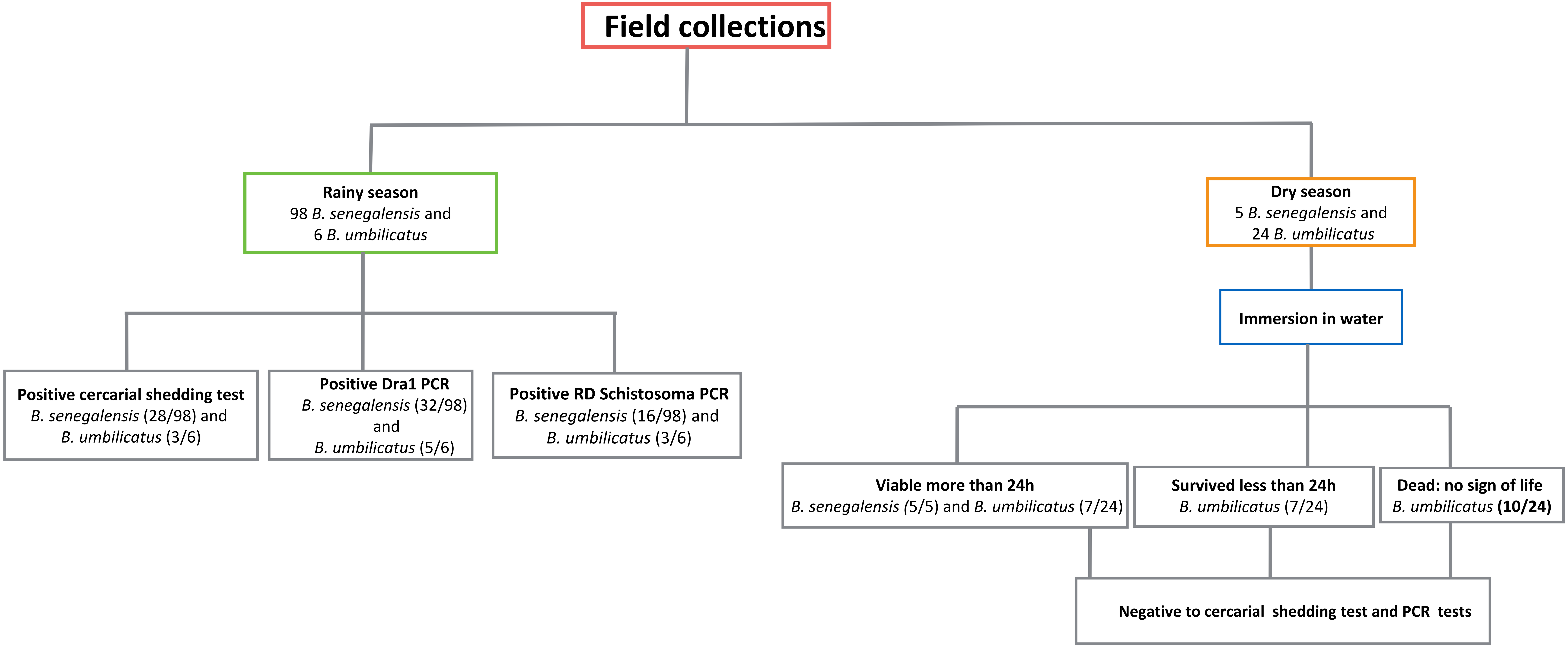

Figure S1. Description of the procedure used to collect molluscs during the dry and rainy seasons, to assess their viability and their infestation by *S. haematobium*.
